# Runoff Forecasting Using Machine-Learning Methods: Case Study in the Middle Reaches of Xijiang River

**DOI:** 10.3389/fdata.2021.752406

**Published:** 2022-02-04

**Authors:** Lu Xiao, Ming Zhong, Dawei Zha

**Affiliations:** ^1^Department of Land Resources and Environment, School of Geography and Planning, Sun Yat-sen University, Guangzhou, China; ^2^Southern Marine Science and Engineering Guangdong Laboratory (Zhuhai), Zhuhai, China; ^3^Pearl River Water Resources Research Institute, Guangzhou, China

**Keywords:** streamflow, water level, forecast, machine learning, wavelet neural network (WNN), generalized regression neural network (GRNN)

## Abstract

Runoff forecasting is useful for flood early warning and water resource management. In this study, backpropagation (BP) neural network, generalized regression neural network (GRNN), extreme learning machine (ELM), and wavelet neural network (WNN) models were employed, and a high-accuracy runoff forecasting model was developed at Wuzhou station in the middle reaches of Xijiang River. The GRNN model was selected as the optimal runoff forecasting model and was also used to predict the streamflow and water level by considering the flood propagation time. Results show that (1) the GRNN presents the best performance in the 7-day lead time of streamflow; (2) the WNN model shows the highest accuracy in the 7-day lead time of water level; (3) the GRNN model performs well in runoff forecasting by considering flood propagation time, increasing the Qualification Rate (*QR*) of mean streamflow and water level forecast to 98.36 and 82.74%, respectively, and illustrates scientifically of the peak underestimation in streamflow and water level. This research proposes a high-accuracy runoff forecasting model using machine learning, which would improve the early warning capabilities of floods and droughts, the results also lay an important foundation for the mid-long-term runoff forecasting.

## Introduction

Runoff forecasting is the foundation of water resource management, deployment, and efficient utilization. It is of great significance to reservoir operation, water resource emergency scheduling, hydro-power generation, and irrigation management decisions (Niu et al., 2018). The river runoff is sensitive to various factors, such as catchment response times and the accuracy of meteorological forecasts with time variability and uncertainty (Lima et al., 2016). Furthermore, it is more difficult to forecast accurately when extreme climatic events occur. The establishment of hydrological models provides important support for runoff forecasting. The runoff process is simulated and forecasted from the perspective of the physical mechanism. However, hydrological model driving relies on the input of a large amount of meteorological data and watershed characteristics parameters. The forecasting process is relatively complicated, and its accuracy is limited to the accuracy and completeness of the data (Nourani, 2017).

With the evolution of big data, runoff forecasting methods become more and more diversified. The research craze for artificial intelligence based on big data has risen. Compared with the traditional hydrological models, the machine-learning models show the advantages of high accuracy, high efficiency, and convenient application so that it has been widely used in runoff forecasting and achieved better forecasting results. The major machine-learning models are applied to runoff forecasting, including artificial neural networks (ANNs), support vector machine (SVM), support vector regression (SVR), and neuro-fuzzy (Mosavi et al., 2018). Badrzadeh et al. (2015) applied four different types of ANNs to forecast real-time floods at Casino station on Richmond River, Australia. Tongal and Booij (2018) developed a simulation framework by coupling a baseflow separation method to three machine-learning methods and discussed performances of models in simulation and forecasting of streamflow regarding model types, input structures, and catchment dynamics in detail. Shortridge et al. (2016) utilized multiple regression and machine-learning approaches to simulate monthly streamflow in five highly seasonal rivers in the highlands of Ethiopia and compare their performance in terms of predictive accuracy, error structure and bias, model interpretability, and uncertainty when faced with extreme climate conditions. Guo et al. (2011) proposed an improved SVM model with adaptive insensitive factors to predict monthly streamflow. Yaseen et al. (2016) explored the potential of the extreme learning machine (ELM) method for forecasting monthly streamflow discharge rates in the Tigris River, Iraq and ELM showed better forecasting performance compared with SVR and the generalized regression neural network (GRNN) models.

The Xijiang River is the longest mainstream of the Pearl River. To investigate the runoff mechanism in the context of climate change, lots of studies have been conducted on projecting hydrological processes and responses in the Xijiang River basin. Wu et al. (2015) investigated the changes in hydrological drought frequency over the Xijiang River basin through an analysis of daily streamflow data observed at major hydrological stations along the river. Yuan et al. (2017) established a modeling chain framework to project the future hydrological changes in the Xijiang River basin and found that extreme low flow would undergo a considerable reduction in the future, indicating that drought risk in the Xijiang River basin was expected to increase significantly. Zhu et al. (2019) analyzed the correlation between the monthly streamflow and the monthly rainfall in Xijiang River through several correlation test methods and clarified that the changes of the monthly discharge are still controlled by natural precipitation variations in Xijiang's fluvial system. With the frequent occurrence of extreme hydrological events caused by climate change and the increasing impact of human activities on natural river runoff, the hydrological process in the Xijiang River basin becomes more random and complicated. Therefore, it is of great significance to carry out high precision runoff forecasting for grasping the future flood and drought conditions of the whole Pearl River basin and ensuring the coordination of water resources.

In this study, a combination of hydrological data and meteorological factors was used as input parameters, and the four different machine-learning models, including backpropagation (BP) neural network, GRNN, ELM, and wavelet neural network (WNN) models, were applied for the forecast of mean streamflow and water level in the 7-day lead time. Moreover, to improve forecast accuracy, the flood propagation mechanism was considered. The objectives of this study are as follows: (1) to propose a more reliable runoff forecasting model; (2) to improve the accuracy and efficiency of runoff forecasting; and (3) to explore the relationship between flood propagation mechanism and runoff in the basin. These findings are expected to provide a more accurate guidance for the early warning of floods and droughts.

## Materials

### Study Area

The Xijiang River is the largest river in Guangxi, China. The total area of the river basin in Guangxi is 20.21 × 10^4^ km^2^, accounting for 85.39% of the total land area of Guangxi. The river basin area above Wuzhou station is 32.70 × 10^4^ km^2^, accounting for 92.88% of the total area of the whole Xijiang River basin. The runoff in the basin is unevenly distributed throughout the year. The annual wet season is from April to September, the streamflow accounts for about 78% of the whole year; the dry season is from October to March of the next year, the streamflow accounts for about 22% of the whole year correspondingly. The mean streamflow of the driest month usually occurs from December to February of the following year, mostly in January.

**Figure 1:**
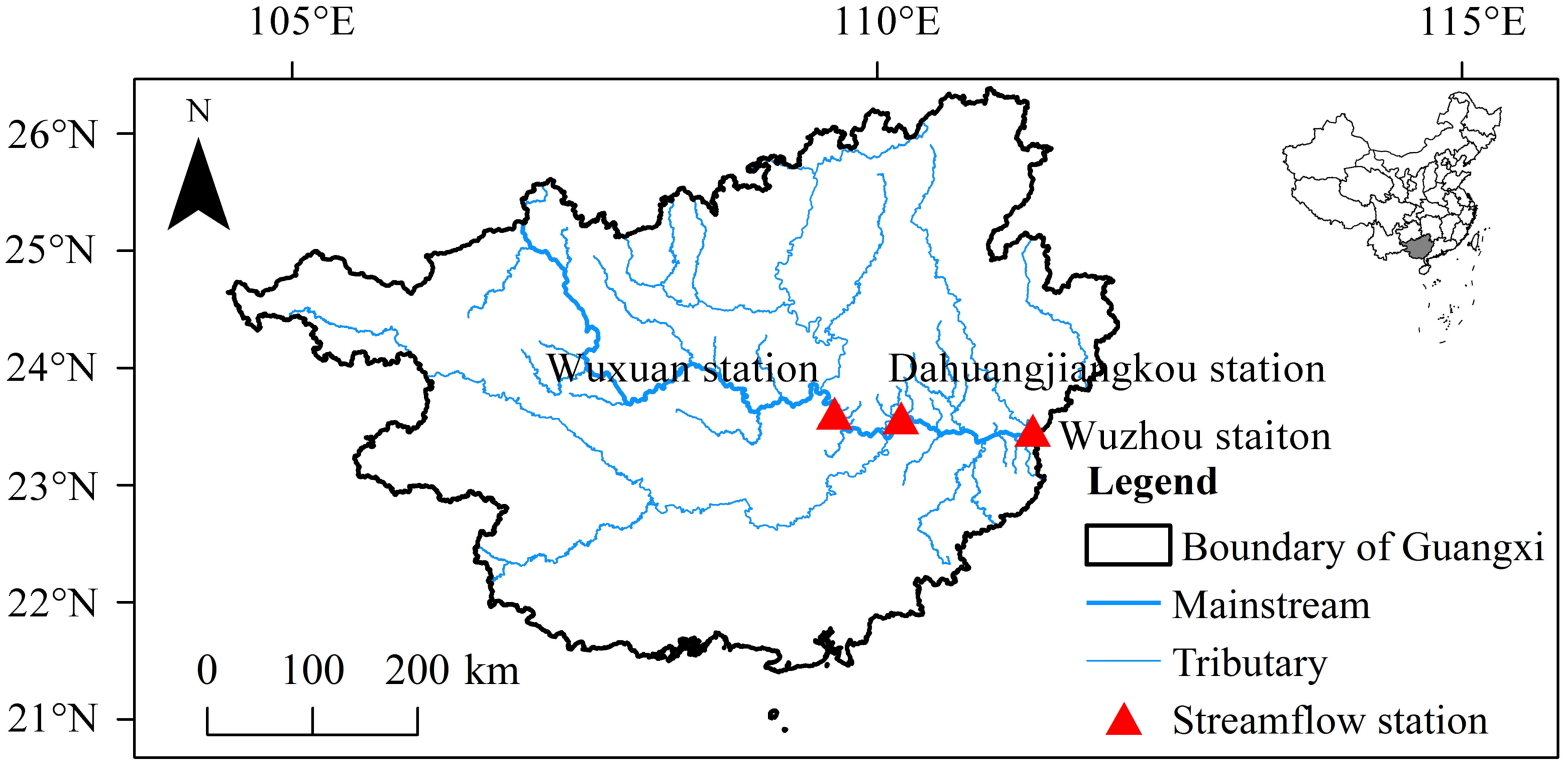
Map of the study area.

The study area was chosen in this research is the Wuxuan-Wuzhou reaches of the Xijiang River in Guangxi with a total length of about 247 km that includes Qianjiang, Xunjiang, and Xijiang River sections. The hydrographic stations from upstream to downstream are Wuxuan station, Dahuangjiangkou station, and Wuzhou station, respectively (Figure 1).

### Data Collection

In this study, daily time series mean streamflow and water level data from 2009 to 2019 measured at Wuxuan station, Dahuangjiangkou station, and Wuzhou station were utilized. The Wuzhou meteorological data were collected from China Meteorological Data Service Center (http://data.cma.cn) that includes daily precipitation (P), average air pressure (PRS), average temperature, mean water vapor pressure (WVP), mean relative humidity (RH), and maximum wind speed (U_max_). After preprocessing operations, such as interpolation, filling, and deletion, the data during 2009–2017 were selected for training and the remainder during 2018–2019 for testing.

## Methodology

The ANN is a technology based on intelligence imitating signal processing in the human brain. It is used as a black-box model with the ability to learn and find out non-linear relationships between the system inputs and outputs. ANNs can efficiently deal with correlation problems when physical processes are not understood or are very complex (Pliego Marugan et al., 2018). The generalization capability of ANN allows it to process unseen data more quickly and simply after learning using a few measured data sets. BP neural network, GRNN, ELM, and WNN models belong to four different types of ANNs (Elsheikh et al., 2019; Lee et al., 2019). These models have been fully used in runoff forecasting, which proves their applicability in accurate prediction (Modaresi et al., 2018; Mosavi et al., 2018; Yaseen et al., 2018; Zhang et al., 2018; Pradhan et al., 2020).

### Backpropagation Neural Network

Backpropagation neural network is a kind of multi-layer forward neural network based on BP. As a typical machine-learning algorithm of ANN, BP neural network architecture includes an input layer, hidden layers, and output layer. Each layer i+s is composed of several neurons (nodes), the output value of each node is determined by the input value, function, and threshold value. The learning process of the BP neural network includes two processes (Bisoyi et al., 2019): information forward propagation and error **back propagation**. In the forward propagation process, the input information is transmitted from the input layer to the output layer through the hidden layers, and the output value is compared with the expected value after the transfer function operation. If there is an error, the error propagates back and returns along the original connection path. Reduce the error by modifying the weight of each layer of neurons layer by layer, and loop until the output result meets the accuracy requirements (Hameed et al., 2017).

The node number of the hidden layer can be determined by Zhang et al. (2018):


(1) $$\begin{array}{l} l<\sqrt{\left(m+n\right)+a} \end{array}$$

Where *l* is the node number of implicit layer; *m* is the node number of output layer; *n* is the node number of input layer; *a* is a constant with any value of 1–10. The optimal value of *l* is determined by trial calculation. The training parameters were assigned as follows: the learning rate is 0.01, the allowable biggest step of the training is 5,000, and the minimum error was set to 10^−5^.

### Generalized Regression Neural Network

Generalized regression neural network is a kind of radial basis neural network, which has strong non-linear mapping ability, flexible network structure, high fault tolerance, and robustness. It is suitable for solving non-linear problems. GRNN has better performance than traditional radial basis function (RBF) networks in terms of approximation ability and learning speed. The network converges to the optimized regression surface with more samples accumulated, and also has better simulation results when processing fewer samples (Li et al., 2013).

Generalized regression neural network model structure consists of the input layer, pattern layer, summation layer, and output layer. The procedure of the GRNN can be represented as (Cigizoglu and Alp, 2006):

If *f* (*x, y*) represents the known joint continuous probability density function of a vector random variable *x* and a scalar random variable *y*, the conditional mean of *y* given *X* (also called the regression of *y* on *X*) is given by


(2) $$\begin{array}{l} Ŷ=E\left(y|X\right)=\frac{\int_{-\infty }^{\infty }yf\left(X,\;y\right)dy}{\int_{-\infty }^{\infty }f\left(X,\;y\right)dy} \end{array}$$

Parzen non-parametric estimation was used to estimate the density function $\hat{f}\left(X,\;y\;\right)$:


(3) $$\begin{array}{l} \hat{f}\left(X,\;y\right)=\frac{1}{{n\left(2\pi \right)}^{\frac{p+1}{2}}\sigma^{p+1}}\overset{n}{\underset{i=1}{\sum }}\exp\left[-\frac{{\left(X-X_{i}\right)}^{T}\left(X-X_{i}\right)}{2\sigma^{2}}\right]\\ \;\exp\left[-\frac{{\left(X-Y_{i}\right)}^{2}}{2\sigma^{2}}\right] \end{array}$$

Substituting $\hat{f}\left(X,\;y\right)$ for Equation (2):


(4) $$\begin{array}{l} Ŷ\left(X\right)=\frac{\sum_{i=1}^{n}Y_{i}\exp\left[-\frac{{\left(X-X_{i}\right)}^{T}\left(X-X_{i}\right)}{2\sigma^{2}}\right]}{\sum_{i=1}^{n}\exp\left[-\frac{{\left(X-X_{i}\right)}^{T}\left(X-X_{i}\right)}{2\sigma^{2}}\right]} \end{array}$$

Ŷ (*X*) is the weighted average of the observed value *Y*_*i*_ of all samples, and the weight factor of each observation is Euclidean distance squared exponent between the corresponding sample *X*_*i*_ and *X*. When the smoothing parameter σ is made large, Ŷ (*X*) is approximately the mean of all the sample dependent variables. On the contrary, the smaller value of σ is, the closer Ŷ (*X*) is to the training sample. When the point to be predicted is included in the training sample set, the predicted value of the dependent variable will be very close to the corresponding dependent variable in the sample. However, once it encounters a point that is not included in the training sample, the prediction effect may be very poor.

### Extreme Learning Machine

Extreme learning machine is an innovative machine-learning algorithm proposed for the deficiency of single-hidden layer feedforward neural network (SLFN). The algorithm randomly generates the continuous weights between the input layer and the hidden layer and the threshold of the hidden layer neurons, and there is no need to adjust during the training process. The unique optimal solution can be obtained only by setting the number of neurons in the hidden layer. Compared with the traditional training method, it has the advantages of fast learning speed and excellent generalization performance (Yaseen et al., 2019; Parisouj et al., 2020; Niu and Fen, 2021).

Mathematically, the ELM model can best be summarized by assuming that there are *N* arbitrarily different training data set samples {(*x*_1_, *y*_1_*y*_1_), …, (*x*_*t*_, *y*_*t*_)}, *t* = 1, 2, …, *N*. In this assumption, *x*_*t*_ is the explanatory variable and *y*_*t*_ is the response variable. *x*_*i*_ ϵ ***R***^*d*^ and *y*_*i*_ ϵ ***R***. The output of SLFN can be expressed as (Huang et al., 2006):


(5) $$\begin{array}{l} \overset{L}{\underset{i=1}{\sum }}B_{i}g_{i}\left(a_{i}\cdot x_{i}+b_{i}\right)=z_{t},\;t=1,\;2,\;\cdots \;,\;N \end{array}$$

where the *L* is hidden nodes number, *g*_*i*_ (*a*_*i*_· *x*_*i*_ + *b*_*i*_) is a hidden layer output function, “Sigmoid” was chosen in this article, *a*_*i*_ is the weight factor connecting input node and the *i*th hidden node, *b*_*i*_ is the bias of the *i*th hidden node, *B*_*i*_ is the weight factor connecting the *i*th hidden node and output node, and *z*_*t*_ is the output of *t*th input.

If the feedforward neural network with *L* hidden nodes can approximate the *N* samples with zero error, there exist *a*_*i*_, *b*_*i*_, and *B*_*i*_ such that:


(6) $$\begin{array}{l} \overset{L}{\underset{i=1}{\sum }}B_{i}g_{i}\left(a_{i}\cdot x_{i}+b_{i}\right)=y_{t},\;t=1,\;2,\;\cdots \;,\;N \end{array}$$

Equation (5) can be simplified as:


(7) $$\begin{array}{l} HB=Y \end{array}$$

*H* is called the hidden layer output matrix of the neural network, the *i*th column of *H* is the *i*th hidden node output with respect to inputs *x*_1_, *x*_2_, …, *x*_N_. In the ELM model, the output weights and deviations can be given randomly, and the hidden layer output matrix *H* becomes a certain matrix so that the training of the feedforward neural network can be transformed into a problem of solving the least square solution of the output weight matrix. The minimum norm square solution of Equation. (7) is:


(8) $$\begin{array}{l} \hat{B}=H^{+}Y \end{array}$$

The *H*^+^ is the Moore-Penrose inverse of hidden layer output matrix *H*.

### Wavelet Neural Network

Wavelet neural network is a multi-layer feedforward network proposed on the basis of wavelet analysis, which integrates the merits of ANN and wavelet analysis (Zhang and Benveniste, 1992). It can not only avoid local optimal fundamentally but also accelerate the learning speed and reduce the training times. In this study, the Morlet wavelet function is used as the mother wavelet, the BP neural network topology is taken as the basis, and the transfer function of the neural network hidden nodes is replaced by the wavelet function. The corresponding weights from the input layer to the hidden layer and the threshold value of the hidden layer are replaced by the scaling factor and translation factor of the wavelet function, respectively (Abghari et al., 2012; Wei et al., 2013).

Assuming that there is a set of input samples *x*_*i*_ (*i* =1, 2, …, *k*), the output of the hidden layer can be constructed using the equation:


(9) $$\begin{array}{l} h\left(j\right)=h_{j}\left(\frac{\sum_{i=1}^{k}\omega_{ij}x_{i}-b_{i}}{a_{j}}\right) \end{array}$$

Where *h*(*j*) is the output of the *j*th hidden layer node, ω_*ij*_ is the weight from the input layer to the hidden layer, *h*_*j*_ is the wavelet function, *a*_*j*_ is the scaling factor of the wavelet function, and *b*_*j*_ is the translation factor of the wavelet function.

The calculation formula of the output layer of WNN is:


(10) $$\begin{array}{l} y\left(k\right)=\overset{l}{\underset{i=1}{\sum }}\omega_{ij}h\left(i\right),\;k=1,\;2,\;\cdots \;,\;m \end{array}$$

Where ω_*ik*_ is the weight from hidden layer to the output layer, *h*(*i*) is the output of the *i*th hidden layer node, *l* is the number of hidden layer nodes, and *m* is the number of output layer nodes.

The gradient learning algorithm is applied to modify the weights, scaling factor, and translation factor. The optimized factors are trained by WNN to obtain the optimal output.

### Verification Model

To evaluate the performance of the four modeling approaches, the following statistical criteria were used:

(1) Mean absolute error (*MAE*):

(11) $$\begin{array}{l} MAE=\frac{1}{N}\overset{N}{\underset{i=1}{\sum }}|O_{i}-P_{i}| \end{array}$$

(2) Deterministic coefficient (*DC*):

(12) $$\begin{array}{l} DC=1-\frac{\sum_{i=1}^{N}\left[{\left(O_{i}-P_{i}\right)}^{2}\right]}{\sum_{i=1}^{N}\left[{\left(O_{i}-\bar{O}\right)}^{2}\right]} \end{array}$$

(3) Correlation coefficient (*R*^2^):

(13) $$\begin{array}{l} R^{2}={\left[\frac{\sum_{i=1}^{N}\left(O_{i}-\bar{O}\right)\left(P_{i}-\bar{P}\right)}{\sqrt{\sum_{i=1}^{N}{\left(O_{i}-\bar{O}\right)}^{2}}\sqrt{\sum_{i=1}^{N}{\left(P_{i}-\bar{P}\right)}^{2}}}\right]}^{2} \end{array}$$

(4) Mean relative error (*MRE*):

(14) $$\begin{array}{l} MRE=\frac{1}{N}\overset{N}{\underset{i=1}{\sum }}\frac{\left|O_{i}-P_{i}\right|}{O_{i}} \end{array}$$

(5) Root mean square error (*RMSE*):

(15) $$\begin{array}{l} RMSE=\sqrt{\frac{1}{N}\overset{N}{\underset{i=1}{\sum }}{\left(O_{i}-P_{i}\right)}^{2}} \end{array}$$

(6) Qualification rate (*QR*):

(16) $$\begin{array}{l} QR=\frac{n}{m}\times 100\% \end{array}$$

Where *O*_*i*_ is the *i*th observation, *P*_*i*_ is the forecasted value of the *i*th model, *N* is the number of samples, $\bar{O}\;$is the average of observed values *O*_*i*_, $\bar{P}$ is the average of model forecasted values *P*_*i*_, *n* is the number of qualified forecasts, and *m* is the total number of forecasts. The closer the value of *MAE* is to 0, the better is the prediction result. When *DC* is between 0 and 1, the closer *DC* is to 1, which implies higher consistency between the forecasted value and the observed value and ultimately reflects in a better model prediction. When *DC* is less than 0, it implies that the forecasted result is undesirable. The closer *R*^2^ is to 1, the higher the degree of correlation between the forecasted values and the observed values. The closer *MRE* is to 0, the better the prediction. The closer *RMSE* is to 0, the smaller the prediction deviation is, and the model is more reliable. The higher the *QR*, the better prediction of the model.

## Results

### Correlation Analysis of Input Parameters

The factors that include mean streamflow before 7 days (*Q*__*t*_−7_), 10 days (*Q*__*t*_−10_), and 15 days (*Q*__*t*_−15_), mean water level before 7 days (*H*__*t*_−7_), 10 days (*H*__*t*_−10_), and 15 days (*H*__*t*_−15_), *P, PRS, T, WVP, RH*, and *U*_max_ of Wuzhou station were used as input parameters of the model to predict the mean streamflow and mean water level. Pearson correlation coefficient between input parameters and mean streamflow and mean water level of Wuzhou station were calculated respectively, and the results are displayed in Table 1. The results show that the mean streamflow, water level, and meteorological factors of Wuzhou station before 7, 10, and 15 days are significantly correlated with the mean streamflow and water level of the day. The runoff forecasting can be reasonably carried out by input of these 8 factors into the machine-learning models.

**Table 1:** Correlation of input parameters with mean streamflow and mean water level of Wuzhou station.

**Parameter**	***Q*__*t*_–7_ (m^3^**·**s^**−1**^)**	***H*__*t*_–7_ (m)**	***Q*__*t*_–10_ (m^3^**·**s^**−1**^)**	***H*__*t*_–10_ (m)**	***Q*__*t*_–15_ (m^3^·s^**−1**^)**	***H*__*t*_–15_ (m)**	***P* (mm)**	***PRS* (hPa)**	***T* (**°**C)**	***WVP* (hPa)**	***RH* (%)**	***U*_max_ (m·s ^**−1**^)**
Streamflow	0.670**	0.698**	0.614**	0.648**	0.565**	0.605**	0.244**	−0.541**	0.473**	0.571**	0.316**	0.237**
Water level	0.704**	0.755**	0.653**	0.705**	0.605**	0.659**	0.247**	−0.603**	0.532**	0.637**	0.353**	0.253**

Pearson correlation coefficient between 0.8 and 1.0, very strong correlation; 0.6–0.8, strong correlation; 0.4–0.6, moderate correlation; 0.2–0.4, weak correlation; 0.0–0.2, very weak correlation or no correlation; “**” represents significant correlation at 0.01 and “*” represents significant correlation at 0.05.

### Determination of the Lead Time

To select a mid-long-term runoff forecasting lead time with satisfactory forecast accuracy, the effects of machine-learning models (i.e., BP, GRNN, ELM, and WNN models) on daily mean streamflow and water level in 7-, 10-, and 15-day lead time were compared. Performance indices in Tables 2, 3 show that the forecast results of mean streamflow and water level in the 7-day lead time are better than those in the 10- and 15-day lead time. Taking the forecast results by BP neural network as an example, the *MAE* values of mean streamflow for 7-, 10-, and 15-day lead time are 1,772.7856, 1,934.0324, and 2,098.2541 m^3^·s^−1^ respectively; *DC* values are 0.2081, 0.0951 and −0.2322, respectively; *R*^2^ values are 0.5224, 0.4541, and 0.4333, respectively; *MRE* values are 0.2630, 0.2995, and 0.3715, respectively; *RMSE* values are 3,036.2640, 3,268.3675, and 3,304.2589 m^3^·s^−1^ respectively; *QR* values are 64.88, 68.86, and 53.50%, respectively. The *MAE* values of mean water level for 7-, 10-, and 15-day lead time are 1.3460, 1.4400, and 1.5976 m, respectively; *DC* values are 0.5205, 0.3759, and 0.2415, respectively; *R*^2^ values are 0.6365, 0.5816, and 0.5125, respectively; *MRE* values are 0.2100, 0.2313, and 0.2712, respectively; *RMSEs* are 1.8897, 1.9926, and 2.1758 m, respectively; *QR* values are 67.22, 63.24, and 60.77%, respectively. It shows that the longer the lead time, the more the model is affected by the uncertainty of input parameters. Therefore, this article discusses the runoff forecasting results of the 7-day lead time.

**Table 2:** Performance indices of Wuzhou station mean streamflow forecast in the 7-, 10-, and 15-day lead time.

	**MAE (m** ^**3**^ **·** **s** ^**−1**^ **)**			**DC**			**R** ^**2**^			**MRE**			**RMSE (m** ^**3**^ **·** **s** ^**−1**^ **)**			**QR (%)**		
	**7-day**	**10-day**	**15-day**	**7-day**	**10-day**	**15-day**	**7-day**	**10-day**	**15-day**	**7-day**	**10-day**	**15-day**	**7-day**	**10-day**	**15-day**	**7-day**	**10-day**	**15-day**
BP	1,772.7856	1,934.0324	2,098.2541	0.2081	0.0951	−0.2322	0.5224	0.4541	0.4333	0.2630	0.2995	0.3715	3,036.2640	3,268.3675	3,304.2589	64.88	68.86	53.50
GRNN	1,783.1870	1,888.3839	2,067.9450	0.5082	0.4338	0.3811	0.5138	0.4497	0.3848	0.2657	0.2767	0.3451	3,066.2337	3,290.1598	3,439.9025	71.06	75.31	61.45
ELM	1,940.0608	2,102.5341	2,165.5322	0.4763	0.4122	0.3705	0.5008	0.4420	0.4051	0.3156	0.3696	0.3741	3,164.0892	3,352.1745	3,469.1480	56.24	51.03	55.28
WNN	1,778.2273	2,129.8711	2,097.3720	0.4940	0.3928	0.3556	0.5003	0.3932	0.3688	0.2951	0.3705	0.3576	3,110.2468	3,407.3199	3,510.0276	59.81	50.62	56.10

**Table 3:** Performance indices of Wuzhou station mean water level forecast in the 7-, 10-, and 15-day lead time.

	***MAE*** **(m)**			***DC***			*****R*** ^**2**^**			***MRE***			***RMSE*** **(m)**			***QR*** **(%)**		
	**7-day**	**10-day**	**15-day**	**7-day**	**10-day**	**15-day**	**7-day**	**10-day**	**15-day**	**7-day**	**10-day**	**15-day**	**7-day**	**10-day**	**15-day**	**7-day**	**10-day**	**15-day**
BP	1.3460	1.4400	1.5976	0.5205	0.3759	0.2415	0.6365	0.5816	0.5125	0.2100	0.2313	0.2712	1.8897	1.9926	2.1758	67.22	63.24	60.77
GRNN	1.3499	1.4714	1.5761	0.6077	0.5458	0.4713	0.6167	0.5588	0.4781	0.2178	0.2409	0.2614	1.9068	2.0518	2.2138	66.12	63.65	65.98
ELM	1.3590	1.4842	1.5871	0.5948	0.5637	0.4687	0.6162	0.5823	0.5001	0.2142	0.2505	0.2729	1.9379	2.0111	2.2192	65.71	60.08	60.22
WNN	1.2725	1.5224	1.5850	0.6401	0.5255	0.4644	0.6412	0.5483	0.4662	0.2017	0.2632	0.2589	1.8264	2.0972	2.2282	69.68	63.79	64.61

### Streamflow Forecast Results of Wuzhou Station by Machine-Learning Model

By comparing all studies of the daily mean streamflow of Wuzhou station forecasting methods (i.e., BP, GRNN, ELM, and WNN models), the forecasting accuracy indices in the 7-day lead time are presented in Table 2. It shows that the four models have a certain forecasting ability for the mean streamflow of Wuzhou station, with *MAE* ranging between 1,772 and 1,941 m^3^·s^−1^, *DC* ranging between 0.20 and 0.50, *R*^2^ ranging between 0.50 and 0.53, *MRE* ranging between 0.26 and 0.32, *RMSE* ranging between 3,036 and 3,165 m^3^·s^−1^ and *QR* ranging between 56 and 72%. In general, comparing the *R*^2^ values of each model, the evaluation accuracy has reached more than 0.50 and the difference is unobvious. But GRNN has the highest *DC* and *QR* values (*DC* = 0.5138, *QR* > 70%) and smaller errors in terms of *MAE, MRE*, and *RMSE* suggesting that the forecasting performance is better.

**Figure 2:**
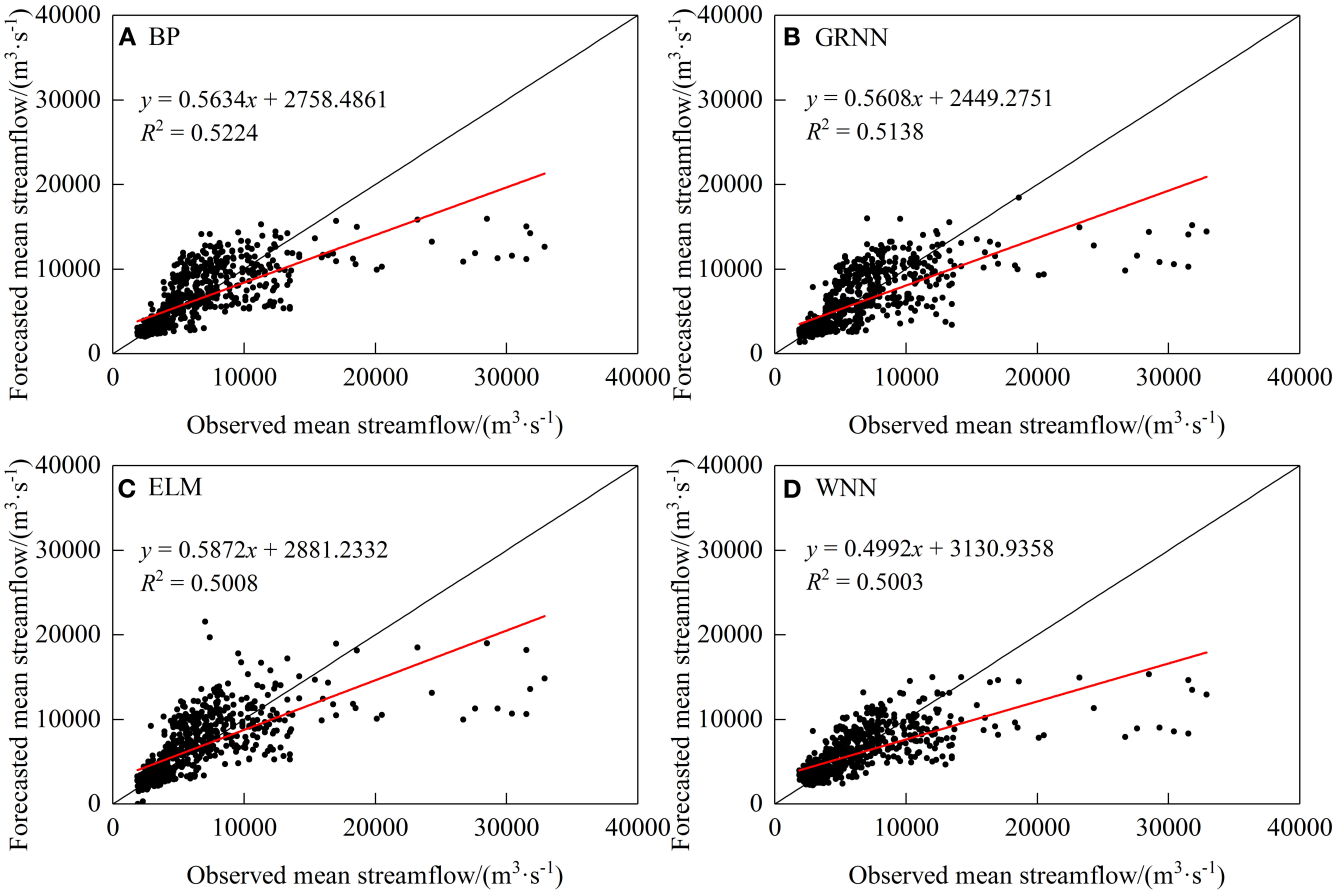
Scatter plots of observed and simulated mean streamflow.

**Figure 3:**
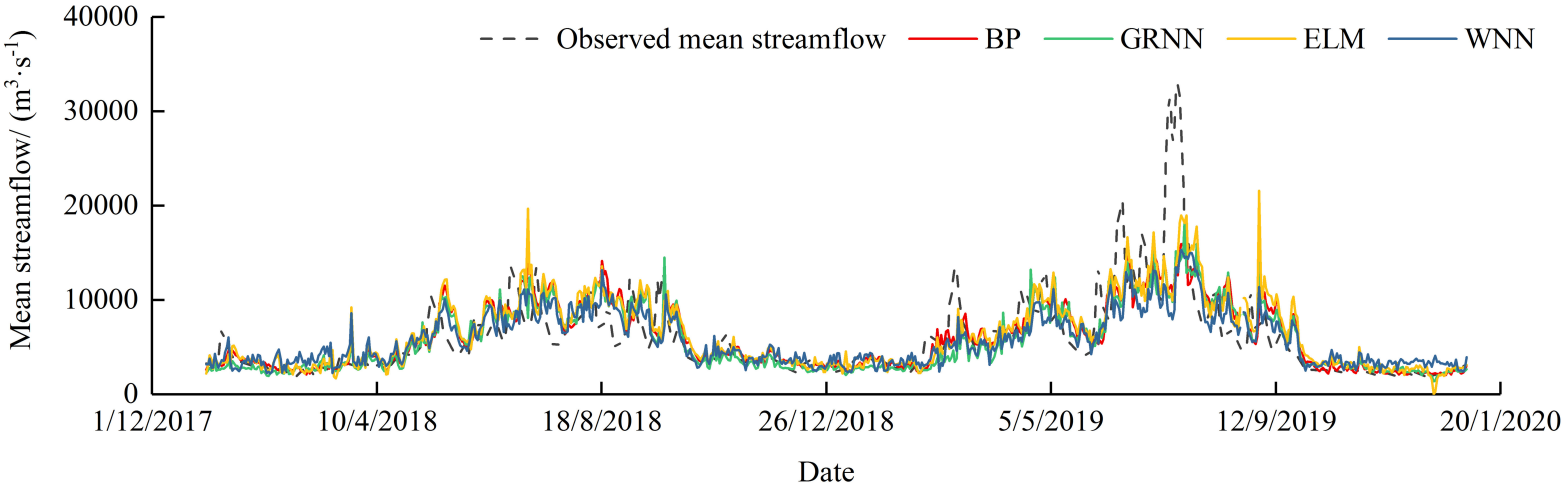
Hydrographs of observed and simulated mean streamflow.

To understand the forecast performance of each model in more detail and intuitively, the scatter plots of the linear regression between forecasted and observed streamflow (Figure 2) and hydrographs (Figure 3) are displayed. Based on the graphical presentations in Figures 2, 3, the four models perform better in the case of low flow values, but regarding the medium flow values, they are overestimated; for the high-flows values, these are underestimated. Obviously, it is difficult for these models to predict the extreme peak flow validly. The reason may be the probability of extreme flood events is low in the period of study, the models are unable to learn such events well.

### Water Level Forecast Results of Wuzhou Station by Machine-Learning Model

Backpropagation, GRNN, ELM, and WNN models were used to forecast the daily mean water level of the Wuzhou station. The forecasting accuracy indices in the 7-day lead time are presented in Table 3. In general, the forecast accuracy of the four models on the mean water level is better than that on the streamflow, with *MAE* ranging between 1.27 and 1.36 m, *DC* ranging between 0.52 and 0.65, *R*^2^ ranging between 0.61 and 0.65, *MRE* ranging between 0.20 and 0.22, *RMSE* ranging between 1.82 and 1.94 m, and *QR* ranging between 65 and 70%. The WNN model shows the smallest error with the highest *DC*, *R*^2^, and *QR* values, which are 0.6401, 0.6412, and 69.68%, respectively.

**Figure 4:**
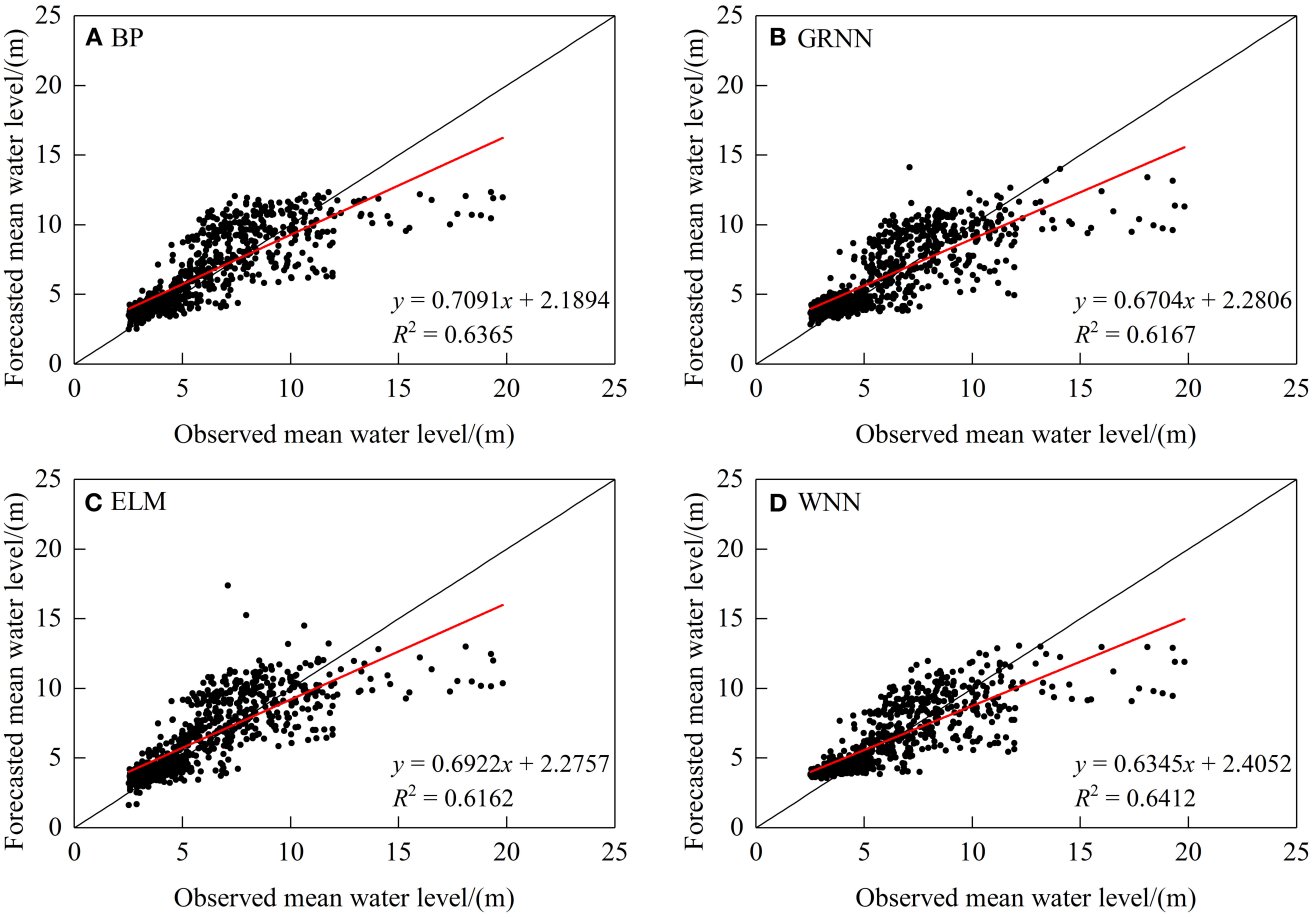
Scatter plots of observed and simulated mean water level.

**Figure 5:**
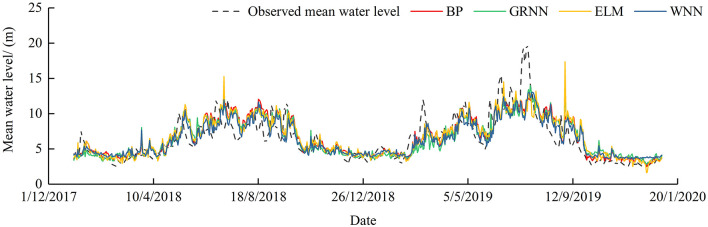
Hydrographs of observed and simulated mean water level.

Figures 4, 5 illustrate the scatter plots of the linear regression between forecasted and observed water level and hydrographs. Similar to the streamflow forecast results, the four models show better performance in the case of medium and low water levels, but significantly underestimate in the case of high water level.

### Forecast Results of Considering the Flood Propagation Time

According to the analysis of Sections Determination of Lead Time, Streamflow Forecast Results of Wuzhou Station by Machine Learning Model, and Water Level Forecast Results of Wuzhou Station by Machine Learning Model, deviations still exist in the forecast of the streamflow and water level of Wuzhou station by meteorological and corresponding hydrological data in the 7-day lead time. There are problems, such as underestimation of the flood peak flow and water level in extreme flood events and lagging of the flood peak forecast. To improve the accuracy of forecasting, the streamflow and water level of Wuzhou station on the day were predicted considering the relationship between upstream and downstream flood propagation.

Wuxuan, Dahuangjiangkou, and Wuzhou stations are important hydrological control stations in the mainstream of Xijiang River. Based on the observed flood data of each station for a series of years, the distance between Wuxuan station and Dahuangjiangkou station is about 104 km, the flood propagation time is about 12 h; the distance between Dahuangjiangkou station and Wuzhou station is about 143 km, the flood propagation time is about 30 h. Therefore, the streamflow and water level data before 2 days at Wuxuan station and the data before 1 day at Dahuangjiangkou station were selected as input parameters to forecast the data of Wuzhou station.

**Figure 6:**
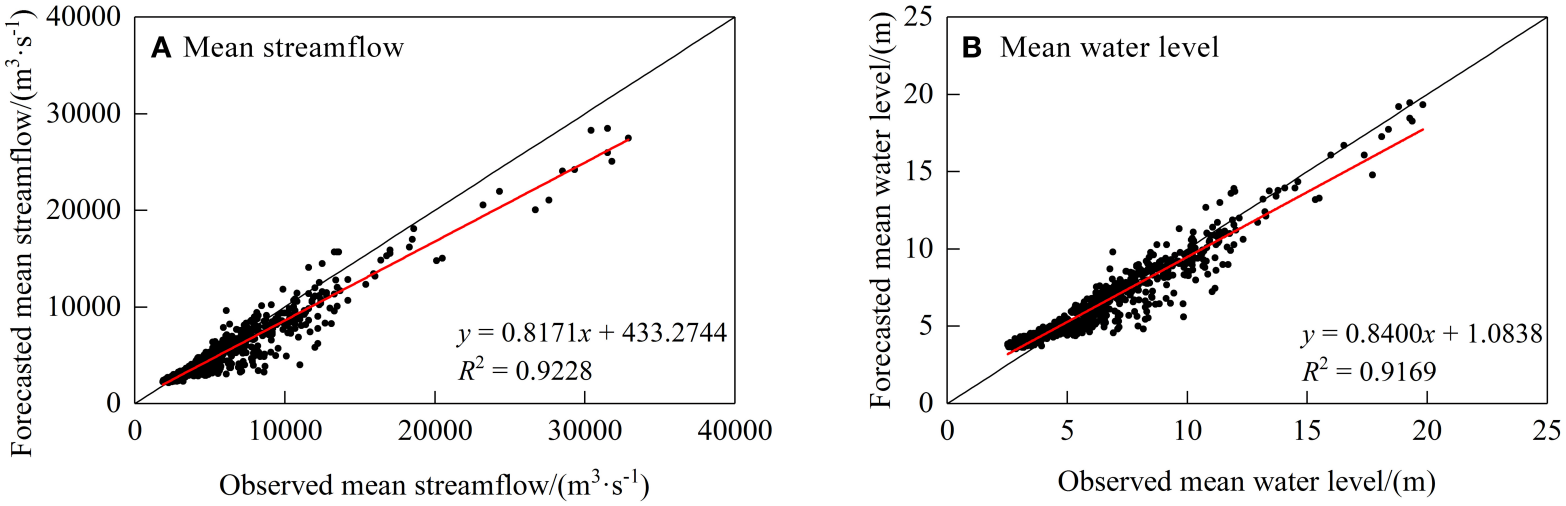
Scatter plots of Wuxuan–Dahuangjiangkou–Wuzhou stations mean streamflow and water level observed and simulated by GRNN.

**Figure 7:**
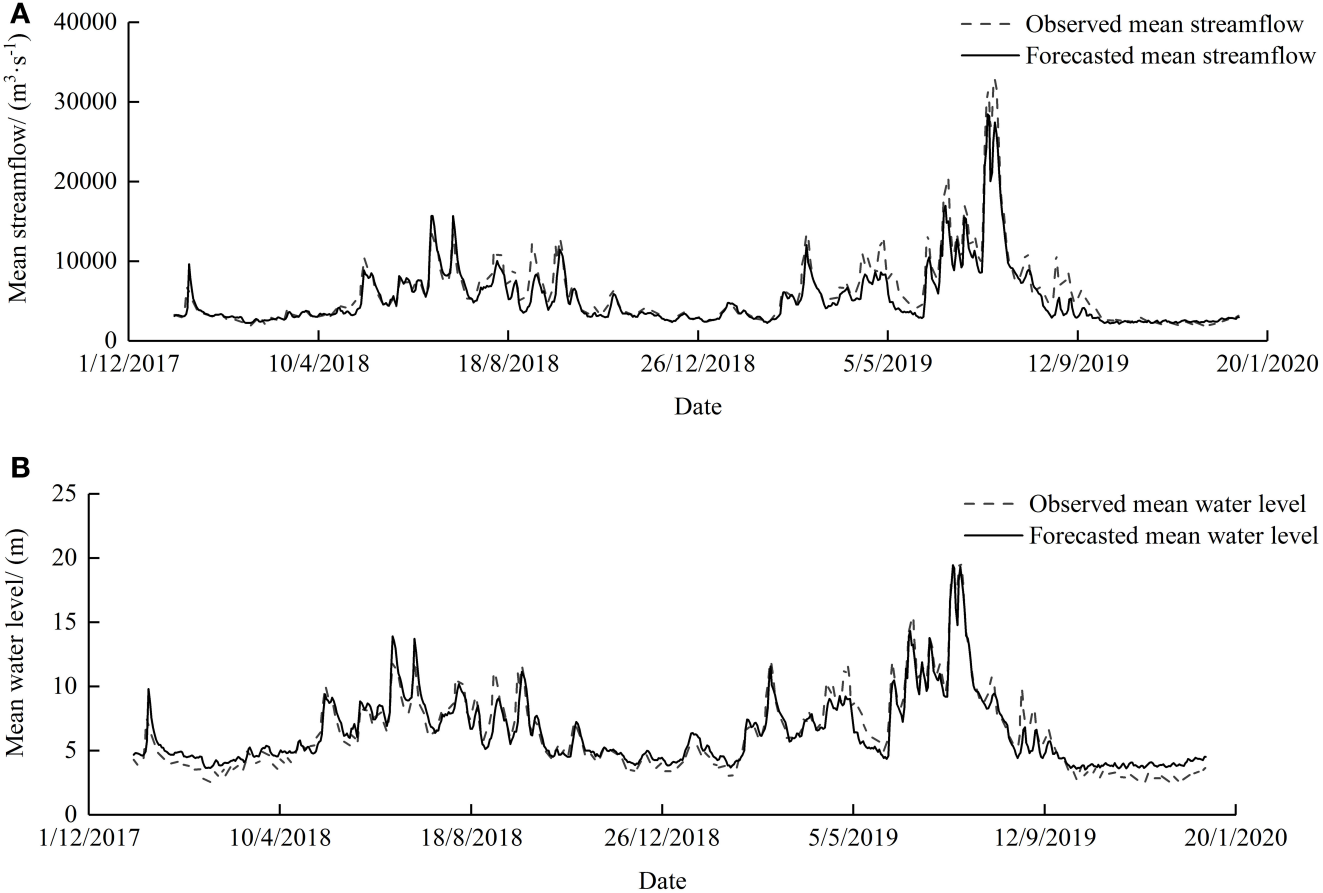
Hydrograph of Wuxuan–Dahuangjiangkou–Wuzhou stations mean streamflow **(A)** and water level **(B)** observed and simulated by GRNN. GRNN, generalized regression neural network.

The analysis indicates that the prediction performance of GRNN is more precise compared to other models, thus GRNN was used for further research. The forecast results are shown in Table 4. GRNN has a satisfying forecast in streamflow and water level with *DC* of 0.8884 and 0.9099, respectively; *R*^2^ of 0.9228 and 0.9169, respectively; and *QR* of 98.36 and 82.74%, respectively. Observed and forecasted streamflows and water level values using the GRNN models are shown in Figures 6, 7. It is evident that there is a significant linear correlation between the forecasted and observed results, which improves the accuracy of high flow and high water level by only inputting hydrological data. Considering the relationship in flood propagation time between upstream and downstream, this method has high accuracy and convenient application, but the shortage is that the lead time is too short to satisfy mid-long term forecasting at present.

**Table 4:** Performance indices of Wuxuan–Dahuangjiangkou–Wuzhou stations mean streamflow and water level forecast by GRNN.

	**MAE**	**DC**	**R^**2**^**	**MRE**	**RMSE**	**QR (%)**
Mean streamflow	895.9491 (m^3^·s^−1^)	0.8884	0.9228	0.1302	1,459.9038 (m^3^·s^−1^)	98.36
Mean water level	0.7117 (m)	0.9099	0.9169	0.1346	0.9134(m)	82.74

## Conclusion

In this study, four different machine-learning methods were utilized to forecast the mean streamflow and water level, including BP, GRNN, ELM, and WNN. Taking Wuzhou station of Xijiang River as a case study, the performances of different models were compared. Furthermore, considering the flood propagation time, the upstream Wuxuan station and the Dahuangjiangkou station streamflow and water level data were used as input parameters to runoff forecasting. The major findings are as follows:

1. GRNN model performs the best on the streamflow forecasting of Wuzhou station in the 7-day lead time, with *DC* = 0.5082, *R*^2^ = 0.5138, and *QR* = 71.06%.
2. WNN model shows the best prediction effect on the water level of Wuzhou station in the 7-day lead time, with *DC* = 0.6401, *R*^2^ = 0.6412, and *QR* = 69.68%. Overall prediction results can meet the accuracy requirements (>60.0%), but it is difficult to make an accurate prediction for extreme events.
3. Considering the relationship between upstream and downstream flood propagation, the accuracy of the machine-learning method is improved significantly. The GRNN model was used for streamflow forecasting with *MAE* of 895.9491 m^3^·s^−1^, *DC* of 0.8884, *R*^2^ of 0.9228, *MRE* of 0.1302, *RMSE* of 1,459.9038 m^3^·s^−1^, and *QR* of 98.36%, and the water level forecasting with *MAE* of 0.7117 m, *DC* of 0.9099, *R*^2^ of 0.9169, *MRE* of 0.1346, *RMSE* of 0.9134 m, and *QR* of 82.74%. This method effectively solved the problem of underestimation in the case of high flow and high water level.

There are still several aspects that can be improved in this study. As revealed in this article, optimizing the structure of machine-learning models to improve the efficiency and accuracy of forecasting and extending the lead time for runoff forecasting utilizing the relationship between upstream and downstream flood propagation is waiting for further research.

## Data Availability Statement

The original contributions presented in the study are included in the article/supplementary material, further inquiries can be directed to the corresponding author/s.

## Author Contributions

MZ: supervision and project administration. LX: model development. DZ: data collection and processing. All authors were involved in the production and writing of the manuscript. All authors contributed to the article and approved the submitted version.

## Funding

The research was funded by the National Key Research and Development Program of China (Grant No. 2021YFC3001000), and the Innovation Group Project of Southern Marine Science and Engineering Guangdong Laboratory (Zhuhai) (Grant No. 311021018).

## Conflict of Interest

The authors declare that the research was conducted in the absence of any commercial or financial relationships that could be construed as a potential conflict of interest.

## Publisher's Note

All claims expressed in this article are solely those of the authors and do not necessarily represent those of their affiliated organizations, or those of the publisher, the editors and the reviewers. Any product that may be evaluated in this article, or claim that may be made by its manufacturer, is not guaranteed or endorsed by the publisher.
